# Premature Mortality and Health Inequality among Adult New Yorkers with Serious Mental Illness

**DOI:** 10.1007/s11524-024-00953-w

**Published:** 2025-02-04

**Authors:** Iva Magas, Christina Norman, Ashwin Vasan

**Affiliations:** 1 Formerly With New York City Department of Health and Mental Hygiene, NY, NY USA; 2 New York City Department of Health and Mental Hygiene, NY, NY USA

**Keywords:** Mental health, SMI mortality, Standardized mortality ratios

## Abstract

Although numerous studies have documented excess mortality and health inequality among individuals with serious mental illness (SMI), none has been done among individuals in a large, diverse urban setting, such as New York City (NYC). We used referral data for adults aged 18 and older referred to the NYC Department of Health and Mental Hygiene public mental health services between January 2004 and December 2018 and matched it to the NYC death registry. Age at death, leading causes of death, years of potential life lost (YPLL), and standardized mortality ratios (SMRs) were calculated for this population. We found individuals with SMI in NYC died at younger ages and had higher rates of YPLL compared to the total population (147.4 YPLL vs. 66.8 YPLL per 1000 population). Age and gender-adjusted SMRs show these individuals have more than twice the mortality rate of the total NYC adult population (overall SMR 2.2 [95% CI 2.1–2.2]). Cause-specific SMRs show an increased risk of death among SMI from diabetes (SMR 2.8 [95% CI 2.4–3.10]), heart disease (SMR 2.7 [95% CI 2.6–2.9]), psychoactive substance use and accidental overdose (SMR 4.5 [95% CI 4.1–4.9]), and suicide (SMR 6.7 [95% CI 6.0–7.4]). Our results highlight the need to implement effective, preventive, and rehabilitative measures that integrate physical and behavioral healthcare services and address upstream drivers of health to achieve health equity and eliminate health disparities. In order to achieve gains in life expectancy, specific considerations for reducing excess mortality in the SMI population must be accounted for.

## Introduction

Multiple studies have documented excess mortality and Years of potential life lost (YPLL) among individuals with serious mental illness (SMI) [1] compared to the general population with all-cause mortality being 2.5–3 times higher in individuals with SMI with reductions in life expectancy up to 20 years [2–6]. These include studies reporting on state or county-wide data and various cohort definitions of individuals with mental illness. However, no such studies have been conducted among individuals living in a large and diverse urban setting such as New York City (NYC) where there are many healthcare facilities, a large public hospital system, and an extensive public transportation system to facilitate getting to and from medical care, potentially making healthcare more accessible than in many parts of the US to individuals with SMI.

Per the latest available estimates, the prevalence of SMI has stayed relatively stable at around 5% of the adult population in the US and in NYC, where there are about 297,000 adults living with SMI [7, 8]. With life expectancy increasing in the general population since the mid-twentieth century, differences between the mortality of those with SMI and that of the general population have not diminished over time [9]. There is increasing evidence that individuals with SMI experience disparities in mortality outcomes and are dying prematurely from both natural and unnatural causes of death [2–6]. The COVID-19 pandemic reinforced this.

Studies from New York City and elsewhere show that individuals with SMI are at higher risk of COVID-19-related mortality than individuals without an SMI [10, 11]. Different risk factors are thought to play a role in premature death among people with SMI including individual factors (e.g., the severity of disorder and behavioral risk factors), health-care system factors (e.g., deficiencies in access to care or information), and social determinants of health (e.g., socio-economic position, stable housing, educational opportunities, access to healthy foods and healthcare) [12, 13].

In NYC, people with SMI are more likely than individuals without an SMI to have lower household income, be unemployed or out of the workforce, have lower educational attainment, and have two or more chronic disease diagnoses. They are also more likely to report they did not get needed medical care in the past year, despite being just as likely to be insured and have a primary care doctor [14].

The goal of the present study was to measure excess mortality among adults with SMI referred to mental health services for individuals in NYC with high service needs from 2004 to 2018 compared to the total adult NYC population. We examined and compared the average age at death, leading causes of death, and years of potential life lost (YPLL) and calculated standardized mortality ratios (SMRs) to compare the extent of premature mortality among those with SMI and the total adult NYC population.

## Methods

### Study Population

This study includes adults ages 18 and older referred to the NYC Department of Health and Mental Hygiene (DOHMH) Single Point of Access (SPOA) services between January 2004 and December 2018 (a 15-year period all years for which SPOA data was available). The SPOA program at NYC DOHMH connects eligible people with SMI to mental health services such as Assertive Community Treatment, Forensic Assertive Community Treatment, Intensive Mobile Treatment, or non-Medicaid Care Coordination [15]. Data for all eligible SPOA referrals were entered into the referral registry, and cases were included in the study whether or not the individual received services over the study period. SMI was determined through both a psychiatric evaluation and a psychosocial evaluation conducted by a psychiatrist or nurse practitioner. Diagnoses include, but are not limited to, schizophrenia, bipolar disorder, and/or major depressive disorder.

### Data Sources and Matching

Data for 43,533 de-duplicated SPOA referrals were matched to the NYC Vital Statistics (NYC VS) death registry to identify deaths for the 15-year referral period from January 2004 through December 2018. Probabilistic matching of the two databases was performed using individuals’ identifiable demographic information (first name, last name, sex, birth date, social security, and Medicaid numbers when available) followed by some manual inspection of the results to eliminate false positives. For the 5481 individuals that died during the study period, demographic information (sex, race/ethnicity), date of death, and the main cause of death were obtained from individuals’ death certificates. The main cause of death is based on the information available on the death certificate as determined by the Chief Medical Examiner and coded using the International Classification of Diseases, 10th revision (ICD 10) [16]. Data on leading causes of death for the 2004–2018 adult NYC population were obtained from NYC Vital Statistics annual mortality data. For the calculation of SMRs, 2004–2018 NYC DOHMH population estimates data were used for the denominator population in this study.

### Ethics

The study was reviewed by the ethical committee of NYC DOHMH, the Institutional Review Board (IRB). The study did not involve interactions or interventions with living individuals and was exempt under the federal regulations 45 CFR §46.101(d)(4)(ii).

### Measures and Analyses

Descriptive analyses for 43,533 individuals referred to SPOA services between 2004 and 2018 were performed to provide context for the study. For the 5481 individuals that died during the study period, frequency distributions were used to describe causes of death, and the mean age at death was calculated to determine whether there were differences in cause and age at death among the SMI and total population.

### Years of Potential Life Lost (YPLL)

YPLL measure represents the number of years a person would have lived if they had not died before the predetermined age of 75 years; hence, it gives more weight to deaths occurring among younger people [17]. We calculated gender-specific average YPLL among the SMI and the total population for all years of the study combined to estimate and compare the average YPLL in both populations. We then calculated the YPLL rate for individuals with SMI and the general population to account for the different population sizes and demonstrate the impact of YPLL on the SMI population compared to the general population.

### Standardized Mortality Ratios (SMRs)

SMRs were used to describe the relative risk of mortality among SMI individuals in comparison to the total population by dividing the observed number of deaths in the SMI cohort by the expected number of deaths derived from the age and sex-matched all-cause and cause-specific mortality found in the total adult NYC population. All presented SMRs were standardized by sex and 5-year age groups. Due to a small number of deaths among those under 20 (three deaths), we excluded those from all SMR calculations. An SMR greater than 1.0 indicates that the relative risk of death for an SMI individual is higher than that of the general population [18, 19]. All mortality metrics were aggregated to show the 2004–2018 average and were performed using SAS Enterprise Guide 7.2 software.

## Results

### Population and Individual Characteristics

Table 1 shows the demographic characteristics of all referred (43,533) and deceased (5481) SMI individuals. The mean (± SD) and median ages at referral were 41.9 (± 14.3) and 42, respectively, with 42% of referred individuals being female and 58% male. The majority (62%) of the study population were from minority racial/ethnic backgrounds, with 40% of eligible referees being non-Hispanic Black, 19% Hispanic/Latino, 4% Asian/Pacific Islander, and 22% non-Hispanic White. It is worth noting that race and/or ethnicity information was missing for 11% of referees. During the 15-year study period, 13% of all referred individuals died.

**Table 1:** Characteristics of referred/eligible and deceased SMI individuals, NYC DOHMH, 2004–2018

Cohort characteristics	All SMI eligible referrals		Deceased SMI referrals	
	*N*	%	*N*	%
	**43,533**	–-	**5481**	**13%**
**Sex**				
Female	18,382	42%	2344	43%
Male	25,135	58%	3137	57%
**Age at referral/death**				
Mean	41.9	± 14.3	58.3	± 13.6
Median	42.0		59.0	
**Age Gr**				
18–24	6193	14%	59	1%
25–34	8598	20%	271	5%
35–44	9339	22%	448	8%
45–54	10,555	24%	1181	22%
55–64	6319	15%	1781	33%
65 +	2529	6%	1741	32%
**Race/ethnicity**				
Asian	1785	4%	134	2%
Black	17,247	40%	2141	39%
Latino/a	8034	19%	1063	19%
Other/unknown	1970	5%	201	4%
White	9713	22%	1939	35%
Missing	4784	11%	3	0%

### YPLL Before Age 75

Table 2 compares the gender-specific age at death and YPLL difference for individuals with SMI and the total NYC population. The average mean ± SD age at death for decedents with SMI was 58.3 ± 13.6 years (60.5 ± 13.4 years for females and 56.6 ± 13.5 for males), while the average mean ± SD age at death for deceased adult individuals in the total NYC population was 73.5 ± 16.8 years (77.0 ± 15.9 years for females and 69.8 ± 17.0 for males) during the years of the study period.

**Table 2:** YPLL before age 75 among deceased SMI and total adult NYC population, 2004–2018 average

*2004–2018 average*	Deceased SMI referrals						NYC adult population					
	Total		Female		Male		Total		Female		Male	
	M	SD	M	SD	M	SD	M	SD	M	SD	M	SD
Age at death	58.3	± 13.6	60.5	± 13.4	56.6	± 13.5	73.5	± 16.8	77	± 15.9	69.8	± 17.0
YPLL before age 75	19.4	± 11.6	17.8	± 11.0	20.5	± 11.9	16.6	± 12.5	15.4	± 11.8	17.4	± 12.9
YPLL rate per 1000	147.4 potential years lost per 1000 population						66.8 potential years lost per 1000 population					

In addition, the average YPLL for those that died before the age of 75 was calculated and compared between the SMI and the total adult NYC population. We found the average YPLL for all causes of death for SMI decedents was 19.4 ± 11.6 years (17.8 ± 11.0 years for females, and 20.5 ± 11.9 for males), while the average YPLL for deceased individuals in the total NYC population was 16.6 ± 12.5 years (15.4 ± 11.8 years for females, and 17.4 ± 12.9 for males) during the years of the study period.

To adjust for population size and make a more meaningful comparison, we calculated the rate of YPLL for the two populations. We found the average YPLL rate for the general adult NYC population was 66.8 years of potential life lost per 1000 population aged 18–74 in NYC, while the average YPLL rate for deceased individuals with SMI was over two times higher at 147.4 years of potential life lost per 1000 population aged 18–74 during the 2004–2018 study period.

### Leading Causes of Death

The distribution of leading causes of death and the cause-specific average age at death among individuals with SMI are shown in Table 3. Heart disease (32%) and malignant neoplasm (15%) were the top two leading causes of death, comparable to the total adult NYC population [20, 21]. By contrast, deaths due to psychoactive substance use and accidental overdose (7%) and suicide (6%) were more represented in the SMI population than in the total adult NYC population. Deaths due to psychoactive substance use and accidental overdose accounted for 2% of all deaths in NYC, and deaths due to suicides accounted for 1% [20, 21].

**Table 3:** Average age at death by top 5 causes of death among SMI and total NYC population, 2004–2018

Top 5 causes of death for people with serious mental illness (SMI)	Deceased SMI referrals			NYC adult population		
	% of total	Age at death		% of total	Age at death	
		*M*	SD		*M*	SD
Diseases of heart	32%	62.4	± 11.9	36%	79.0	± 9.7
Malignant neoplasms (cancers)	15%	61.7	± 10.1	25%	70.4	± 10.2
Psychoactive substance use and accidental overdose	7%	47.9	± 10.8	2%	45.3	± 11.9
Intentional self-harm (suicide)	6%	43.8	± 13.6	1%	47.0	± 14.7
Diabetes mellitus	5%	57.0	± 12.9	3%	72.3	± 10.5

While suicide fatalities (43.8 ± 13.6 years for SMI and 47.0 ± 14.7 for the NYC population) and deaths due to substance use and accidental drug overdose (47.9 ± 10.8 years for SMI and 45.3 ± 11.9 for the NYC population) had the youngest cause-specific mean age at death in both cohorts, we found premature death among individuals with SMI largely comes from natural and preventable causes of death. When compared to the total NYC population, people with SMI had average ages of death that were 13 to 17 years lower, on average, for the following causes: disease of the heart (62.4 ± 11.9 vs. 79.0 ± 9.7 years), diabetes (57.0 ± 12.9 vs. 72.3 ± 10.5 years), chronic lower respiratory diseases (63.7 ± 10.3 vs. 76.5 ± 10.8 years), and influenza and pneumonia (63.6 ± 11.2 vs. 80.0 ± 10.6 years, respectively).

### SMRs

Table 4 presents the aggregated and top five cause-specific SMRs for all years of the study combined. Overall, both females and males with SMI in NYC have over two times higher risk of dying than the total adult NYC population (SMR 2.2 [95% CI 2.1–2.2]). The highest cause-specific SMR was observed for suicide deaths (SMR 6.7 [95% CI 6.0–7.4]), followed by the SMRs for individuals that died due to use of or poisoning by psychoactive substance (SMR 3.6 [95% CI 3.2–3.9]). In addition, SMRs associated with heart disease (SMR 2.7 [95% CI 2.6–2.9]) and SMRs associated with diabetes (SMR 2.8 [95% CI 2.4–3.1]) in the SMI cohort were almost threefold of the total adult NYC population. The only cause-specific SMR comparable to the total adult NYC population was for deaths due to malignant neoplasms (slightly elevated SMR 1.1 [95% CI 1.0–1.2]).

**Table 4:** SMRs by the leading cause of death and gender among SMI individuals who were 20 years or older at the time of death, 2004–2018

Cause of death	All		Female		Male	
	SMR	95% CI	SMR	95% CI	SMR	95% CI
**All**	2.2*	**(2.1–2.2)**	2.4*	**(2.3–2.5)**	1.9*	**(1.8–2.0)**
Diseases of heart	2.7*	(2.6–2.9)	3.1*	(2.9–3.3)	2.4*	(2.3–2.6)
Malignant neoplasms (cancers)	1.1*	(1.0–1.2)	1.2*	(1.1–1.3)	1.0*	(0.9–1.1)
Psychoactive substance use and accidental overdose	3.6*	(3.2–3.9)	5.3*	(4.4–6.2)	2.8*	(2.4–3.1)
Intentional self-harm (suicide)	6.7*	(6.0–7.4)	10.7*	(8.8–12.7)	4.9*	(4.3–5.6)
Diabetes mellitus	2.8*	(2.4–3.1)	3.5*	(2.9–4.1)	2.2*	(1.8–2.6)

*p < .0001

Furthermore, gender-specific SMRs reveal adult females with SMI in NYC have a greater relative risk of dying than adult males for all causes of death (female SMR 2.4 [95% CI 2.3–2.5] vs. male SMR 1.9 (95% CI 1.8–2.0]). This difference was the highest for suicide deaths (female SMR 10.7 [95% CI 8.8–12.7] vs. male SMR 4.9 [95% CI 4.3–5.6]), and accidental drug poisoning deaths (female SMR 5.3 [95% CI 4.4 −6.2] vs. male SMR 2.8 [95% CI 2.4–3.1], respectively).

### Age at Death and YPLL by Race/Ethnicity

Table 5 compares the average age at death and YPLL difference for SMI individuals and the total NYC population by individuals’ racial and ethnic background recorded in the Vital Statistic data (death certificate).

**Table 5:** Average age at death and years of potential life lost before age 75 among SMI and adult NYC population by race/ethnicity, 2004–2018

Race/ethnicity	Average age at death		Average age gap	YPLL		YPLL gap
	SMI	NYC population		SMI	NYC population	
Black	56.4	69.1	12.7 years	20.5	17.6	2.9 years
Hispanic/Latino	56.4	69.3	12.9 years	20.9	18.2	2.7 years
White	62	77.6	15.6 years	17.3	14.8	2.5 years

Life expectancy for Asians and Pacific Islanders is not displayed because the required single-year-of-age population denominators are too small to produce reliable estimates

Similar to the pattern among the general NYC adult population, the average age at death among White adults with SMI was higher compared to Black and Hispanic/Latino adults with SMI. However, White adults with SMI experienced a larger mortality gap (expressed as difference in average age at death) with the general adult White population compared to the mortality gap experienced by Black and Hispanic/Latino adults with SMI. White adults with SMI die 15.6 years earlier on average than the general population of White adults. This difference in the mortality gap between the general adult population and adults with SMI is 12.9 years for Hispanic/Latino and 12.7 years for Black individuals.

Comparing the YPLL among the SMI and the general adult population in NYC by race and ethnicity, we found a difference of 2.5 years for White, 2.7 for Hispanic/Latino, and 2.9 years for Black SMI individuals.

## Discussion and Conclusion

Our findings support the literature on excess mortality among individuals with SMI. When compared to the general adult NYC population, we found that adults with SMI in NYC had an average age at death that was 15 years younger than adults in the general NYC population (16.5 years younger age at death for females with SMI, and 13.2 years younger age at death for males with SMI).

Individuals with SMI are largely dying of the same causes as the general adult population with the top two causes of death being the same: heart disease and cancers. Deaths due to the use of or poisoning by psychoactive substances and suicide are more represented in the SMI population, although less common in both groups. Consistent with previous research on people with SMI, premature death among SMI in NYC is largely due to natural and preventable causes of death, such as heart disease and diabetes [2, 22] that could potentially be prevented if detected and treated in a timely manner.

Lutterman et al. report in the Sixteen State Study that the SMI population lost 25–30 years of potential life, on average. However, they did not calculate YPLL for individuals without SMI, therefore overestimating the true difference in YPLL between the SMI and the general population [22]. We found an approximately 3-year gap (2.8 overall, 2.4 for females, and 3.1 for males) in YPLL between the SMI and the total NYC adult population. This difference is similar to that reported by Piatt et al. [4] and smaller than that reported by Dembling et al. [23] comparing the YPLL among the SMI and the general population (4.2-year gap and an 8.8-year gap, respectively). This suggests that the gaps in excess mortality did not diminish over time and that pertinent determinants are not being addressed despite the development of health-promoting interventions designed for people with SMI. Our findings are also consistent with limited research reporting individuals with SMI had excess mortality, irrespective of their race and ethnicity, compared to the general population [24].

However, due to differences in population size and demographic characteristics of the general adult population and the population of adults with SMI, we believe it is more appropriate to report the YPLL rate rather than YPLL. Our findings indicate the average YPLL rate for the SMI population is two times the YPLL rate of the total adult NYC population.

Furthermore, we found that the relative risk of death for adults with SMI is more than twice that of the total adult NYC population, a finding that is consistent with previous research [25–27]. The relative risk of death was particularly high for suicide deaths and deaths due to the use of or poisoning by psychoactive substances, similar to what has been noted in other studies [3, 4, 25–28].

While the literature reports discrepancies in the sex difference of mortality risk among the SMI population (some studies report a small but significant male excess [26, 29], whereas other studies report either no sex difference [3] or higher mortality ratios in females compared with males [30]), our analyses show the all-cause mortality risk among females with SMI in NYC is higher than all-cause mortality among SMI males in NYC. Furthermore, females with SMI in NYC had a nearly 11-fold higher risk of dying by suicide than did females in the general NYC population.

These findings suggest a need for primary care and mental health providers to routinely screen individuals with SMI for chronic health conditions such as diabetes, hypertension, high cholesterol, and obesity to identify health conditions early in their course when treatment can be most beneficial. In addition, regular screening for tobacco, alcohol, and substance use can help mitigate the negative impact of the use of these substances on patients’ health. Routine screening for depression and suicidal ideation can help identify people at risk of self-harm, which may be particularly critical for females with SMI given their high risk of dying from suicide relative to females in the general population. Lastly, screening for and addressing the upstream drivers of premature mortality such as housing stability, food security, and access to healthcare is critical to improving mortality outcomes among individuals with SMI [12].

In addition to interventions at the individual level, policies that promote better coordination of mental health and primary care services can help reduce barriers to care for individuals with SMI. Creating and supporting policies that promote social determinants of health for people with SMI will help address the upstream drivers of premature mortality [12]. This includes advocating for expanded access to healthcare and mental healthcare, expanding access to affordable housing and supportive housing, and increasing access to postsecondary education.

Lastly, these findings also suggest that investments in psychosocial rehabilitation, like recent investments in clubhouses in NYC [31], may be helpful at scale as they have been shown through supportive community structures to reduce social isolation and thereby increase rates of health promotion and engagement [32, 33].

New York City experienced a dramatic drop in life expectancy of 4.6 years from 2019 to 2020 and despite some improvement is still over 2 years behind the 2019 life expectancy of 82.6 years [34]. COVID-19 explains much of this phenomenon, but rising rates of chronic illnesses, downstream impacts of mental health conditions including suicide, homicide, and overdoses, as well as other factors, are preventing NYC from regaining its lifespan. In response, New York City launched *HealthyNYC* [34], a unified, citywide population health and prevention strategy aimed at reducing the leading causes of overall risk, premature death and inequity, and excess deaths in the city, in order to reach the highest ever life expectancy of 83 years by 2030.

In order to achieve the life expectancy gains outlined in *HealthyNYC* and codified into *NYC Local Law 93—Intro 93-A* [35], specific attention must be paid to reducing excess mortality among vulnerable groups like people with SMI. This would make New York City unique in planning for this marginalized community within central planning around improving citywide population health.

## Strengths and Limitations

We believe our study has numerous strengths. First and foremost, this is the first study to evaluate mortality among SMI in NYC. Second, our cohort includes data for 15 years, providing us with a large enough sample of individuals with SMI who used public mental health services in NYC, and our findings should be generalizable to people with SMI living in other large and diverse urban areas using a similar level of service. This is also one of the few studies to date that looks at mortality outcomes by race and ethnicity among people with SMI.

However, this study has several limitations that need to be considered as well. Death counts of both the SMI population and general population in our study may be undercounted because deaths that occur outside of NYC are not reported to the NYC Vital Statistics. Furthermore, in this study, we did not distinguish between individuals in our SMI cohort who ultimately accepted and received mental health services from those who did not. It is possible that mortality outcomes differ between these groups.

Our findings might not apply to all individuals with SMI in NYC as the sociodemographic characteristics of the SMI cohort referred to mental health services for individuals with high service needs may be different from the characteristics of SMI individuals in NYC that were not referred to these mental health services.

Lastly, due to inadequate data on race and ethnicity in the referral registry, we were not able to examine all mortality metrics by racial and ethnic groups among SMI. The vital statistic death data did not include any missing values for the race and ethnicity of the deceased, but it is possible that there are inaccuracies in this data set as well. We did offer some findings that are consistent with the limited research that has shown that individuals with SMI have excess mortality in comparison to the general population, regardless of their race or ethnicity.

Future work can focus on examining the impact of risk and protective factors on premature mortality of individuals with SMI, including social determinants of health, to better inform policy and programming to improve mortality outcomes among people with SMI.
